# New Tripentone Analogs with Antiproliferative Activity

**DOI:** 10.3390/molecules22112005

**Published:** 2017-11-18

**Authors:** Barbara Parrino, Salviana Ullo, Alessandro Attanzio, Virginia Spanò, Stella Cascioferro, Alessandra Montalbano, Paola Barraja, Luisa Tesoriere, Girolamo Cirrincione, Patrizia Diana

**Affiliations:** Department of Biological, Chemical, and Pharmaceutical Sciences and Technologies (STEBICEF), University of Palermo, via Archirafi 32, 90123 Palermo, Italy; barbara.parrino@unipa.it (B.P.); salviana.ullo@unipa.it (S.U.); alessandro.attanzio@unipa.it (A.A.); virginia.spano@unipa.it (V.S.); stellamaria.cascioferro@unipa.it (S.C.); alessandra.montalbano@unipa.it (A.M.); paola.barraja@unipa.it (P.B.); luisa.tesoriere@unipa.it (L.T.); girolamo.cirrincione@unipa.it (G.C.)

**Keywords:** tripentones, aza-indoles, 8*H*-thieno[2,3-*b*]pyrrolizinones, antitumor activity, proapoptotic agents

## Abstract

Tripentones represent an interesting class of compounds due to their significant cytotoxicity against different human tumor cells in the submicro-nanomolar range. New tripentone analogs, in which a pyridine moiety replaces the thiophene ring originating the fused azaindole system endowed with anticancer activity viz 8*H*-thieno[2,3-*b*]pyrrolizinones, were efficiently synthesized in four steps with fair overall yields (34–57%). All tripentone derivatives were tested in the range of 0.1–100 μM for cytotoxicity against two human tumor cell lines, HCT-116 (human colorectal carcinoma) and MCF-7 (human breast cancer). The most active derivative, with GI_50_ values of 4.25 µM and 20.73 µM for HCT-116 and MCF-7 cells, respectively, did not affect the viability of Caco-2 differentiated in normal intestinal-like cells, suggesting tumor cells as the main target of its cytotoxic action. The same compound was further investigated in order to study its mode of action. Results showed that it did not exert necrotic effects, while induced a clear shift of viable cells towards early apoptosis. Flow cytometric analysis demonstrated that this compound caused cell cycle alteration, inhibiting its progression in S and G2/M phases.

## 1. Introduction

Cancer is one of the most common causes of morbidity representing an important social problem supposed to maintain its primacy after heart and circulatory disorders, with many new cases in the world per year [1,2]. In most cases the prognosis is relatively poor; in fact, although in recent years several improvements in treatment and prevention have been made, the number of new cases is still increasing. For these reasons and considering the fact that chemotherapy represents the most effective strategy to fight cancer, in the recent years many efforts have been made to find new and effective anticancer molecules [3,4,5,6,7,8].

**Chart 1:**
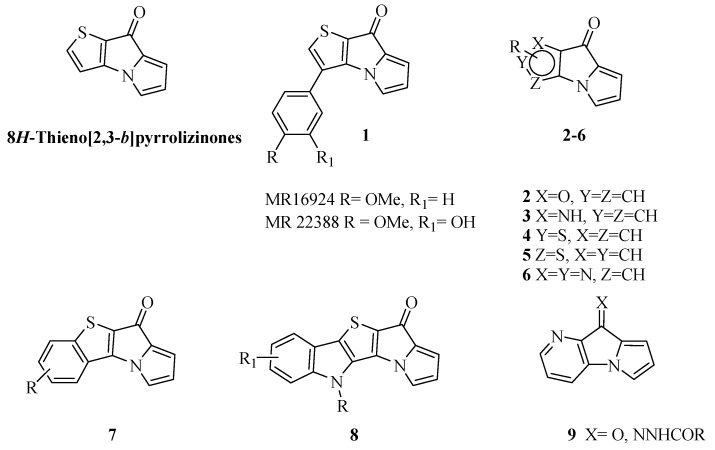
Tripentone analogs.

Pyrrole-fused indoles and related aza-derivatives have emerged as an interesting class of anti-cancer agents endowed with potent activity against different human tumor cell lines and with different mechanisms of action [9,10,11,12,13,14,15,16,17,18,19,20]. In particular, 8*H*-thieno[2,3-*b*]pyrrolizinones, also known as “tripentones” (Chart 1), have been shown to demonstrate significant cytotoxicity against tumor cells in the submicro-nanomolar range [21,22]. Different tripentone analogs have been synthesized; in particular, 3-aryl-8*H*-thieno[2,3-*b*]pyrrolizin-9-ones **1**, tested on 60 tumor cell lines (National Cancer Institute, NCI), showed high cytotoxicity [21] and selectivity against the leukemia subpanel [22]. The most active compound, MR16924, showing IC_50_ values in the submicromolar range, was elected as the lead for the synthesis of different analogs in which the thiophene ring was replaced by bioisosteric analogs such as furan (**2**), pyrrole (**3**), other thiophene isomers (**4**,**5**) [22,23], pyrazole (**6**) [24], or in which benzene (**7**) [25] or the indole ring (**8**) were added [26] (Chart 1). Biological results confirmed the importance of the thienopyrrolizinone core, although many derivatives exhibited interesting cytotoxic properties. The most active compound, MR22388, a thieno[2,3-*b*]pyrrolizin-9-one derivative, showed significant antitumor activity in different human tumor cell lines with IC_50_ values in the submicromolar or nanomolar range. Biological studies performed on this compound demonstrated that it acts as a pro-apoptotic agent, causing cell cycle arrest at the G2/M phase and increasing caspase 3 activity. Further studies, performed in order to clarify its mechanism of action, showed that it causes the inhibition of tubulin polymerization with an IC_50_ value of 2.9 µM, as well as the activation of several kinases including FLT3-ITD [27]. Compound MR22388 was also investigated in vivo, but the results were less exciting than those obtained in the in vitro studies, probably due to the poor bioavailability of the tested drug [22].

Continuing on our ongoing studies on nitrogen heterocyclic systems endowed with antitumor activity [28,29,30,31,32,33,34,35], and considering that hydrazide chains could improve the potency and pharmacokinetic properties of compounds bearing them [36], herein we report the synthesis of new 9*H*-pyrido[2,3-*b*]pyrrolizin-9-one tripentone analogs **9**, in which a pyridine replaces the thiophene ring of 8*H*-thieno[2,3-*b*]pyrrolizinones and in which different carbohydrazine chains were inserted with the aim of endowing them with the proper pharmacokinetic properties responsible for biological activity.

## 2. Results and Discussion

### 2.1. Chemisty

**Scheme 1:**
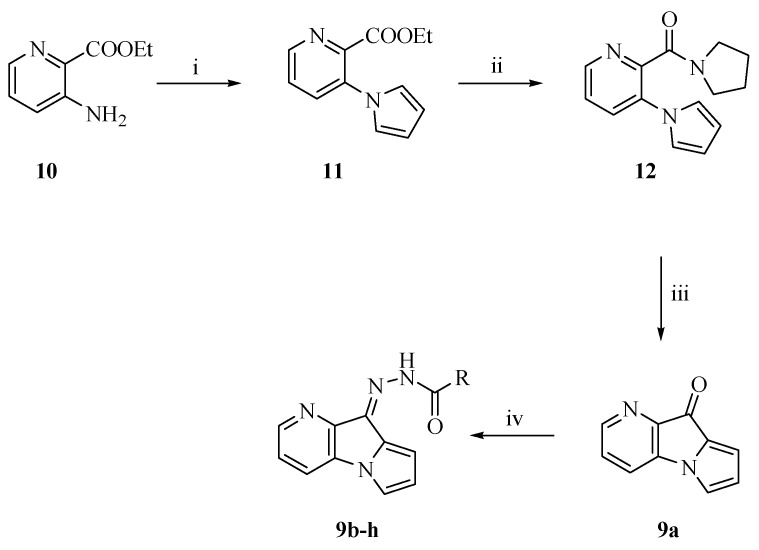
Synthesis of tripentones **9a**–**h**. Reagents and conditions: (i) 2,5-dimethoxytetrahydrofuran, 4-chloropyridine hydrochloride, 1,4 dioxane, r.t., 15 min then **10**, reflux, 18 h, 92%; (ii) Method A: pyrrolidine, reflux, 36 h, 38%; Method B: (a) LiOH, ethanol, reflux 4 h; (b) EDC, HOBt, DIPEA, tetrahydrofuran, r.t., 10 min then pyrrolidine, r.t., 12 h, 88%; (iii) POCl_3_, 70 °C, 6 h, 70%; (iv) RCONHNH_2_, toluene or ethanol, reflux, 24–32 h, 60–93%.

Tripentone compounds were synthesized as described in Scheme 1. The synthetic pathway starts from compound **10**, prepared from the corresponding 3-aminopicolinic acid as described in the literature [37]. The latter was reacted under Clauson-Kaas conditions in the presence of 2,5-dimethoxytetrahydrofuran, 4-chloropyridine hydrochloride in anhydrous 1,4-dioxane at reflux to give, in excellent yields (92%), the pyrrolidine derivative **11,** which was then converted to its corresponding amide **12**. The first attempt was made in refluxing pyrrolidine in a ratio up to 1:42 for 36 h, but unfortunately the desired amide was obtained in low yield (38%). We decided to try a different method via carboxylic acid, using the activating agent of the carboxylic function *N*-(3-dimethylaminopropyl)-*N*′-ethylcarbodiimide hydrochloride (EDC) and hydroxy-benzotriazole (HOBt). The corresponding carboxylic acid was obtained by hydrolysis of the ester group under basic conditions, using lithium hydroxide (LiOH) in ethanol at reflux for 4 h. Subsequent amidation with pyrrolidine, in the presence of EDC, HOBt, and *N,N*-diisopropylethylamine (DIPEA), in tetrahydrofuran at room temperature for 12 h afforded the desired amide **12**, isolated in high yield (88%) and in a shorter time.

Cyclization of the latter was performed by acylation under Vilsmeier-Haack conditions followed by alkaline treatment. Reaction of the amide **12** with phosphorous oxychloride (POCl_3_) afforded an intermediate iminium salt that was subsequently hydrolyzed (NaOH 10%) to give tripentone **9a** isolated in good yield (70%, Table 1). The latter was used for the successive reactions of substitution with suitable heteroaryl carbohydrazide side chains, performed in toluene or ethanol under reflux, to give derivatives **9b**–**h** (60–93%, Table 1).

### 2.2. Biology

#### 2.2.1. Cytotoxic Activity

**Figure 1:**
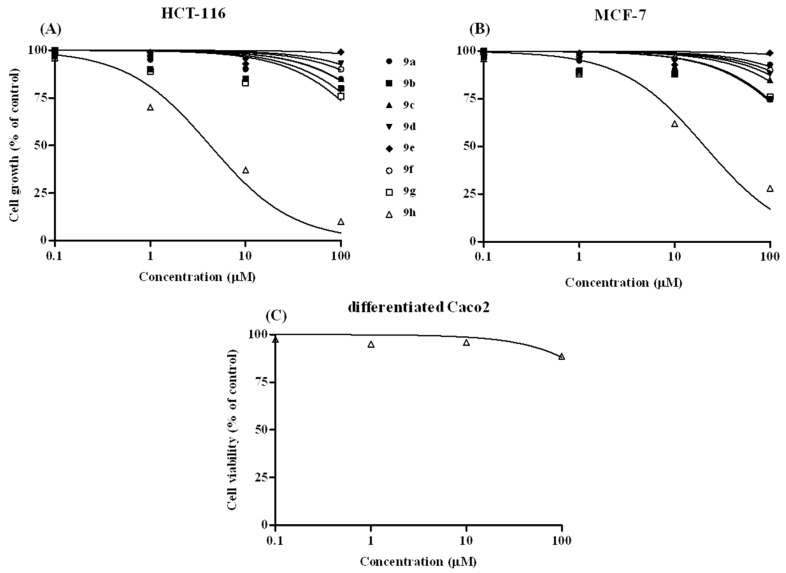
Effect of the synthesized tripentones **9a**–**h** on the growth of human tumor cells HCT-116 (**A**) and MCF-7 (**B**) and of the active **9h** derivative on the viability of intestinal normal-like differentiated Caco-2 cells (**C**). Cells were treated with the compounds and cell viability was measured after 72 h by MTT assay in comparison to cells treated with vehicle alone (control). Values are the means of three separate experiments performed in triplicate.

All synthesized tripentone derivatives **9a**–**h** were tested in the concentration range of 0.1–100 μM for the evaluation of cytotoxicity against two human tumor cell lines, HCT-116 (human colorectal carcinoma) and MCF-7 (human breast cancer), by MTT assay. Compounds **9a**–**g** did not substantially affect the tumor cells viability, whereas **9h** effectively inhibited the growth of both cell lines (Figure 1A,B). Calculated GI_50_ values, i.e., the molar concentration of the compound that inhibits 50% cell growth, were 4.25 ± 0.31 µM and 20.73 ± 1.22 µM for HCT-116 and MCF-7 cells, respectively.

Interestingly, derivative **9h** had no effect on the vitality of Caco-2 differentiated in normal intestinal-like cells, suggesting tumor cells as the main target of its cytotoxic action (Figure 1C).

#### 2.2.2. Cell Death

**Figure 2:**
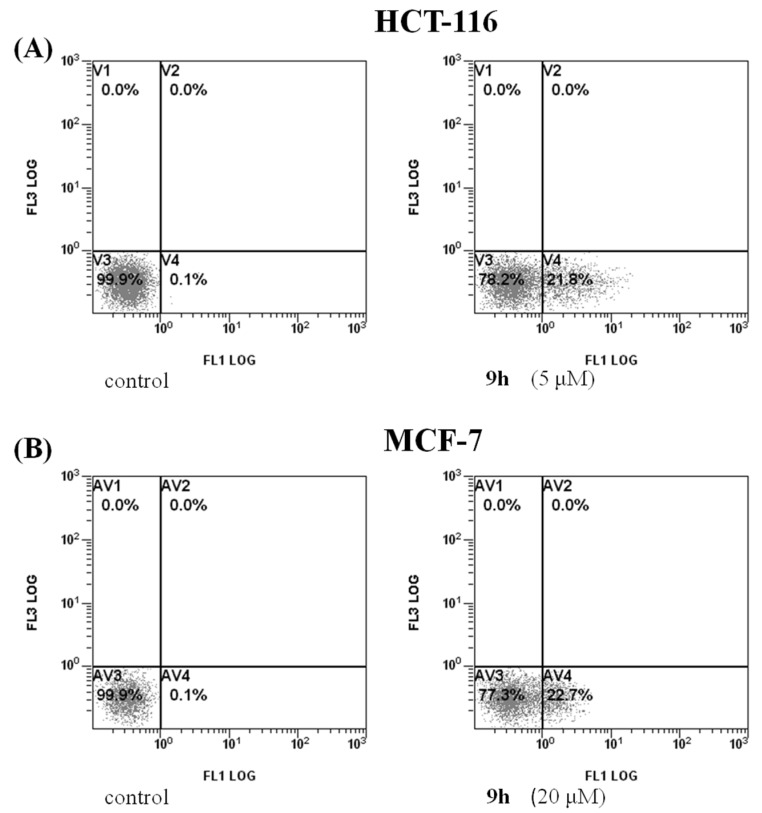
Flow cytometric analysis for the quantification, by annexin V/PI double staining, of **9h** derivative-induced apoptosis in HCT-116 (**A**) or MCF-7 (**B**) cells. Cell monolayers were incubated for 24 h in the absence (control) or presence of the synthesized compound at the GI_50_ value and submitted to double staining with annexin V/PI as reported in the Experimental Section. V3/AV3, viable cells (annexin V−/P−); V4/AV4, cells in early apoptosis (annexin V+/PI−); V2/AV2, cells in tardive apoptosis (annexin V+/PI+); V1/AV1, necrotic cells (annexin V−/PI+). Images are representative of three experiments with comparable results.

To investigate the effects of the inhibition on cell growth (necrosis or apoptosis) of the active synthesized tripentone **9h**, flow cytometry analysis of annexin V-FITC and propidium iodide (PI)-stained HCT-116 and MCF-7 cells was carried out to evaluate the externalization of plasma membrane phosphatidylserine, a reliable marker of cell apoptosis. The concentration chosen for this study was selected taking into account the value of GI_50_ measured in both cell lines. As shown in Figure 2, the tripentone derivative **9h** did not exert necrotic effects, but induced a clear shift of viable cells towards early apoptosis.

#### 2.2.3. Cell Cycle Analysis

**Figure 3:**
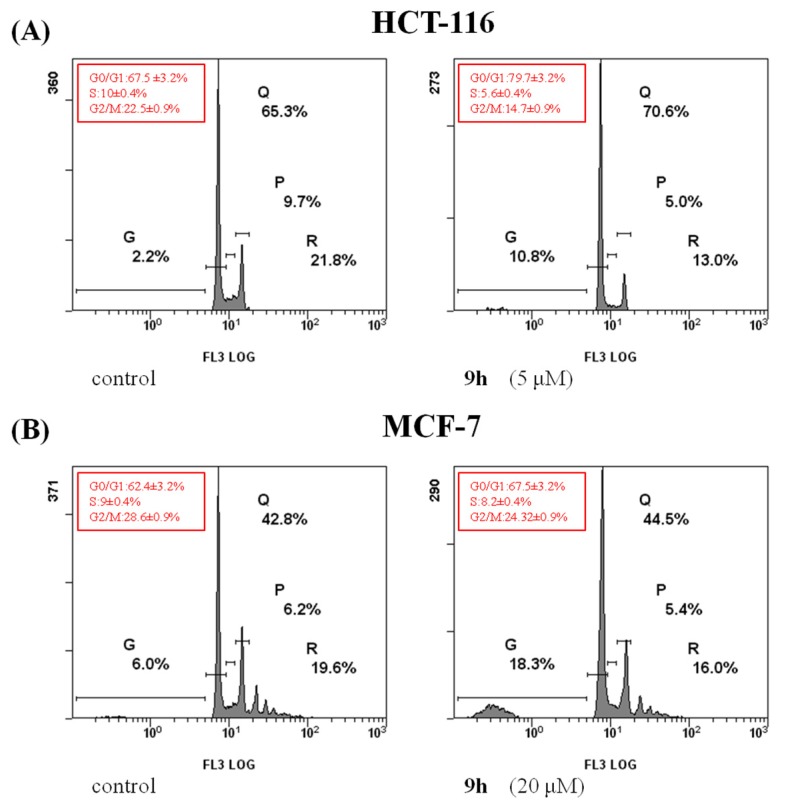
Cell cycle analysis of HCT-116 (**A**) or MCF-7 (**B**) cells treated with compound **9h**. Cell monolayers were incubated in the absence (control) or presence of the compound at the GI_50_ value. After 24 h incubation, propidium iodide-stained cells were submitted to flow cytometric analysis, as reported in the Experimental Section. The percentage of cells in the different phases of the cycle was calculated by Expo32 software. Images are representative of three experiments with comparable results. Inset: percentage of viable cells ±SD in the different phases.

Apoptosis of the tumor cells induced by the synthesized **9h** derivative may be due to abnormal regulation of the cell cycle. To test this possibility, we performed a flow cytometric analysis of PI-stained HCT-116 and MCF-7 cells after 24 h of treatment with the compound at the relevant GI_50_ value. As shown in Figure 3, **9h** caused cell cycle alteration in both tumor cell lines, inducing a block of the viable cells in phase G0/G1 compared to the control and inhibiting the progression of the cell cycle in S and G2/M phases. The appearance of a sub-G1-cell population, which is representative of cells with fragmented DNA, was also evident and consistent with the apoptotic activity of the compound (Figure 3).

These data suggest that tripentone derivative **9h** induces a failure of DNA synthesis, hindering G1/S transition of the cell cycle. Then, different from other tripentones [22], the new synthesized **9h** compound does not appear to be endowed with antitubulin activity, which is responsible for the mitotic failure and arrest in the G2/M phase of the cell cycle.

## 3. Materials and Methods

### 3.1. Chemistry

All melting points were taken on a Büchi-Tottoly capillary (Büchi, Cornaredo, Italy) apparatus and are uncorrected. IR spectra were determined in bromoform with a Shimadzu FT/IR 8400S spectrophotometer (Shimadzu Corporation, Milan, Italy). ^1^H- and ^13^C-NMR spectra were measured at 200 and 50.0 MHz, respectively, in DMSO-*d*_6_ or CDCl_3_ solution, using a Bruker Avance II series 200 MHz spectrometer (Bruker, Milan, Italy). Multiplicity of the ^13^C signals were determined through DEPT spectra. Column chromatography (Sigma Aldrich, Milan, Italy) was performed with Merk silica gel 230–400 mesh ASTM or with Büchi Sepacor chromatography module (prepacked cartridge system). Elemental analyses (C, H, N) were within ±0.4% of theoretical values and were performed with a VARIO EL III elemental analyzer (Elementar, Langenselbold, Germany). Purity of all the tested compounds was greater than 95%, determined by HPLC (Agilent 1100 Series) (Agilent Technologies, Milan, Italy).

#### 3.1.1. Synthesis of Aminopyridine-Carboxylates (**10**)

Experimental procedure and spectroscopic data were in accordance with those reported [37].

#### 3.1.2. Synthesis of Ethyl 3-(1*H*-Pyrrol-1-yl)pyridine-2-carboxylate (**11**)

To a solution of 2,5-dimethoxytetrahydrofuran (0.23 mL, 1.81 mmol) in anhydrous 1,4-dioxane (21.4 mL), 4-chloropyridine hydrochloride (0.27 g, 1.81 mmol) was added and the reaction mixture was stirred at room temperature for 15 min. Compound **10** (0.30 g, 1.81 mmol) was added and the reaction mixture was heated to reflux for 18 h. Upon cooling, the formed precipitate was filtered. The solid was discarded, while the mother liquor containing the title compound was evaporated in vacuo, giving, as pure compound, derivative **11**.

Yield: 92%, light yellow oil; IR: 1696 (CO) cm^−1^; ^1^H-NMR (200 MHz, CDCl_3_) δ: 1.08 (3H, t, *J* = 7.1 Hz, CH_3_), 4.14 (2H, q, *J* = 7.1 Hz, CH_2_), 6.21 (2H, s, H-3′ and H-4′), 6.71 (2H, s, H-2′ and H-5′), 7.35 (1H, dd, *J* = 8.2, 4.7 Hz, H-5), 7.58 (1H, dd, *J* = 8.2, 1.5 Hz, H-4), 8.47 (1H, dd, *J* = 4.7, 1.5 Hz, H-6); ^13^C-NMR (50 MHz, CDCl_3_) δ: 13.8 (q), 62.1 (t), 110.6 (d × 2), 121.6 (d × 2), 125.9 (d), 134.0 (d), 136.1 (s), 146.0 (s), 147.6 (d), 165.5 (s). Anal. Calculated for C_12_H_12_N_2_O_2_ (MW: 216.24): C, 66.65; H, 5.59; N, 12.96%. Found: C, 66.79; H, 5.30; N, 12.76%.

#### 3.1.3. Synthesis of 2-(Pyrrolidin-1-yl)-3-(1*H*-pyrrol-1-yl)pyridine (**12**)

Method A: A solution of **11** (0.30 g, 1.39 mmol) in pyrrolidine (58.38 mmol, 4.8 mL) was heated to reflux for 36 h. Upon cooling, the reaction mixture was concentrated under reduced pressure and the yellow oil was crystallized by diethyl ether and purified by silica gel column chromatography using ethyl acetate as eluent. Yield: 38%.

Method B: To a solution of **11** (0.52 g, 2.41 mmol) in ethanol (73.2 mL), lithium hydroxide (0.29 g, 12.0 mmol) was added and the reaction mixture was heated to reflux for 4 h. After cooling to room temperature, the solvent was removed under reduced pressure. The crude, cooled by adding to ice, was acidified with 6N HCl and extracted with dichloromethane (DCM) (3 × 30 mL), dried over anhydrous Na_2_SO_4_, and evaporated in vacuo. The crude was taken up onto the next step.

To a solution of the crude in tetrahydrofuran (THF) (36.5 mL), hydroxy-benzotriazole (OHBt) (0.36 g, 2.64 mmol), *N,N*-diisoproprylethylamine (DIPEA) (0.68 mL, 2.64 mmol), and *N*-(3-dimethylaminopropyl)-*N′*-ethylcarbodiimide hydrochloride (EDC) (0.34 g, 2.64 mmol) were added and the resulting reaction mixture was stirred at room temperature for 10 min. Pyrrolidine (1.0 mL, 12 mmol) was added and the mixture was stirred at room temperature for 12 h. The solvent was removed under reduced pressure, and an aqueous saturated NaHCO_3_ solution (20.4 mL) was added to the residue. The crude was extracted with ethyl acetate (3 × 30 mL), dried over anhydrous Na_2_SO_4_, filtered, and evaporated in vacuo. It was purified by silica gel column chromatography eluting by DCM:ethyl acetate, 75:25 to give the desired amide. Yield in two steps: 88%.

Yield in two steps: 88%, light yellow powder; m.p.: 117.5–118.0 °C; IR: 1623 (CO) cm^−1^; ^1^H-NMR (200 MHz, DMSO-*d*_6_) δ: 1.74 (4H, quint, *J* = 6.2 Hz, CH_2_ × 2), 2.96 (2H, t, *J* = 6.2 Hz, CH_2_), 3.41–3.34 (2H, m, CH_2_), 6.28 (2H, s, H-3′ and H-4′), 7.04 (2H, s, H-2′ and H-5′), 7.60 (1H, dd, *J* = 8.2, 4.6 Hz, H-5), 7.99 (1H, d, *J* = 8.2 Hz, H-4), 8.56 (1H, d, *J* = 4.6 Hz, H-6); ^13^C-NMR (50 MHz, DMSO-*d*_6_) δ: 23.7 (t), 25.2 (t), 44.9 (t), 46.6 (t), 110.5 (d × 2), 120.9 (d × 2), 124.9 (d), 132.5 (d), 133.2 (s), 146.6 (s), 146.9 (d), 148.6 (s). Anal. Calculated for C_14_H_15_N_3_O (MW: 241.29): C, 69.69; H, 6.27; N, 17.41%. Found: C, 69.87; H, 6.07; N, 17.29%.

#### 3.1.4. Synthesis of 9*H*-Pyrido[2,3-*b*]pyrrolizin-9-one (**9a**)

A solution of pyrrolidinecarboxamide **12** (0.229 g, 0.95 mmol) in phosphorus oxychloride (2.4 mL, 25.65 mmol) was stirred at 70 °C for 6 h. After cooling, the reaction mixture was concentrated to give the iminium salt as a black solid. A 10% aqueous NaOH solution (6.65 mL) was slowly added to the residue and the reaction mixture was heated to 65 °C for 30 min. Upon cooling, the dark crude was extracted with ethyl acetate (3 × 15 mL), dried over anhydrous Na_2_SO_4_, and evaporated in vacuo. It was purified by silica gel column chromatography eluting by DCM:ethyl acetate, 95:5 to give the desired tripentone.

Yield: 70%, yellow solid; m.p.: 187.4–188.4 °C; IR: 1722 (CO) cm^−1^; ^1^H-NMR (200 MHz, DMSO-*d*_6_) δ: 6.44 (1H, m, H-7), 6.96 (1H, d, *J* = 2.5 Hz, H-8), 7.52 (1H, dd, *J* = 7.9, 4.6 Hz, H-3), 7.73 (1H, m, H-6), 7.98 (1H, d, *J* = 7.9 Hz, H-4), 8.39 (1H, d, *J* = 4.6 Hz, H-2); ^13^C-NMR (50 MHz, DMSO-*d*_6_) δ: 115.4 (d), 116.2 (d), 119.0 (d), 122.9 (d), 127.4 (d), 130.2 (s), 139.9 (s), 146.3 (d), 147.9 (s), 177.7 (s). Anal. Calculated for C_10_H_6_N_2_O (MW: 170.17): C, 70.58; H, 3.55; N, 16.46%. Found: C, 70.78; H, 3.35; N, 16.16%.

#### 3.1.5. Synthesis of Substituted 9*H*-Pyrido[2,3-*b*]pyrrolizin-9-ylidenes (**9b**,**d**,**e**,**h**)

To a solution of 9*H*-pyrido[2,3-*b*]pyrrolizin-9-one **9a** (0.05 g, 0.29 mmol) in toluene (4 mL), the opportune heteroaryl carbohydrazide (0.29 mmol) was added. The resulting suspension was refluxed, using Dean-Stark apparatus, for 24–32 h and then chilled overnight. The product was collected by filtration, washed with toluene, and dried under vacuum to afford compound **9e**. In the case of derivatives **9b**,**d**,**h**, the reaction mixture was quenched with a small amount of water, then extracted with DCM (3 × 10 mL), dried over anhydrous Na_2_SO_4_, and evaporated in vacuo. It was purified by silica gel column chromatography eluting by DCM:MeOH, 97:3 (**9b**), DCM (**9d**), and DCM:ethyl acetate, 90:10 (**9h**) to give the desired compound.

*N′-[9H-Pyrido[2,3-b]pyrrolizin-9-ylidene]pyridine-4-carbohydrazide* (**9b**): Conditions: reflux for 24 h. Yield: 69%, light brown powder; m.p.: 244.0–244.4 °C; IR: 3402 (NH), 1683 (CO) cm^−1^; ^1^H-NMR (200 MHz, CDCl_3_) δ: 6.41 (1H, t, *J* = 3.7 Hz, H-7), 6.89 (1H, d, *J* = 3.7 Hz, H-8), 7.10 (1H, d, *J* = 3.7 Hz, H-6), 7.38 (1H, dd, *J* = 7.9, 5.0 Hz, H-4), 7.57 (1H, dd, *J* = 7.9, 1.8 Hz, H-3), 7.88 (2H, dd, *J* = 4.4, 1.8 Hz, H-3′ and H-5′), 8.38 (1H, dd, *J* = 5.0, 1.8 Hz, H-2), 8.85 (2H, dd, *J* = 4.4, 1.8 Hz, H-2′ and H-6′), 14.15 (1H, bs, NH); ^13^C-NMR (50 MHz, CDCl_3_) δ: 109.8 (d), 115.9 (d), 116.3 (d), 117.5 (d), 121.3 (d × 2), 125.1 (d), 130.6 (s), 136.2 (s), 139.7 (s), 140.1 (s), 143.5 (d), 147.5 (s), 150.8 (d × 2), 162.0 (s). Anal. Calculated for C_16_H_11_N_5_O (MW: 289.29): C, 66.43; H, 3.83; N, 24.21%. Found: C, 66.21; H, 3.68; N, 24.36%.

*N′-[9H-Pyrido[2,3-b]pyrrolizin-9-ylidene]furan-2-carbohydrazide* (**9d**): Conditions: reflux for 32 h. Yield: 72%, yellow oil; IR: 3317 (NH), 1680 (CO) cm^−1^; ^1^H-NMR (200 MHz, CDCl*_3_*) δ: 6.39 (1H, t, *J* = 3.6 Hz, H-7), 6.60 (1H, d, *J* = 3.6 Hz, H-8), 6.85 (1H, d, *J* = 3.1 Hz, H-6), 7.08 (1H, d, *J* = 3.2 Hz, H-3), 7.36 (2H, t, *J* = 8.0 Hz, H-3′ and H-5′), 7.55 (1H, dd, *J* = 8.0, 1.3 Hz, H-4′), 7.63 (1H, m, H-4), 8.40 (1H, d, *J* = 3.2 Hz, H-2), 13.95 (1H, bs, NH); ^13^C-NMR (50 MHz, CDCl*_3_*) δ: 109.0 (d), 112.3 (d), 115.4 (d), 115.9 (d), 116.1 (d), 117.1 (d), 124.6 (d), 130.9 (s), 135.9 (s), 138.6 (s), 143.5 (d), 145.0 (d), 147.1 (s), 147.4 (s), 155.2 (s). Anal. Calculated for C_15_H_10_N_4_O_2_ (MW: 278.27): C, 64.74; H, 3.62; N, 20.13%. Found: C, 64.89; H, 3.58; N, 20.07%.

*N′-[9H-Pyrido[2,3-b]pyrrolizin-9-ylidene]thiophene-2-carbohydrazide* (**9e**): Conditions: reflux for 24 h. Yield: 70%, dark yellow powder; m.p.: 163.0–163.7 °C; IR: 3428 (NH), 1656 (CO) cm^−1^; ^1^H-NMR (200 MHz, DMSO-*d*_6_) δ: 6.44 (1H, t, *J* = 3.8 Hz, H-7), 6.75 (1H, dd, *J* = 3.6, 0.8 Hz, H-8), 7.30 (1H, t, *J* = 3.8 Hz, H-6), 7.64–7.58 (2H, m, H-3′ and H-5′), 7.90–7.84 (1H, m, H-4′), 8.00 (1H, dd, *J* = 5.1, 1.2 Hz, H-3), 8.12 (1H, dd, *J* = 8.1, 1.2 Hz, H-4), 8.50 (1H, d, *J* = 5.1, 1.2 Hz, H-2), 13.93 (1H, bs, NH); ^13^C-NMR (50 MHz, DMSO-*d*_6_) δ: 107.5 (d), 115.4 (d), 117.7 (d), 119.1 (d), 125.3 (s), 125.8 (d), 128.2 (d), 129.0 (d), 129.6 (d), 132.8 (s), 135.7 (s), 137.2 (s), 143.9 (d), 146.1 (s), 164.7 (s). Anal. Calculated for C_15_H_10_N_4_OS (MW: 294.33): C, 61.21; H, 3.42; N, 19.04%. Found: C, 61.35; H, 3.28; N, 19.14%.

*N′-[9H-Pyrido[2,3-b]pyrrolizin-9-ylidene]benzohydrazide* (**9h**): Conditions: reflux for 27 h. Yield: 76%, yellow powder; m.p.: 177.2–178.0 °C; IR: 3393 (NH), 1681 (CO) cm^−1^; ^1^H-NMR (200 MHz, DMSO-*d*_6_) δ: 6.44 (1H, t, *J* = 3.3 Hz, H-7), 6.74 (1H, d, *J* = 3.3 Hz, H-8), 7.70–7.53 (5H, m, H-3′, H-4′, H-5′, H-3 and H-6), 7.98 (2H, dd, *J* = 6.2, 1.4 Hz, H-2′ and H-6′), 8.11 (1H, dd, *J* = 8.2, 0.8 Hz, H-4), 8.50 (1H, dd, *J* = 4.8, 0.8 Hz, H-2), 13.90 (1H, bs, NH); ^13^C-NMR (50 MHz, DMSO-*d*_6_) δ: 107.7 (d), 115.4 (d), 117.7 (d), 119.1 (d), 125.7 (d × 2), 127.2 (d), 129.1 (d × 2), 129.7 (s), 132.6 (d), 133.2 (s), 135.7 (s), 138.3 (s), 143.9 (d), 146.2 (s), 162.3 (s). Anal. Calculated for C_17_H_12_N_4_O (MW: 288.30): C, 70.82; H, 4.20; N, 19.43%. Found: C, 70.75; H, 4.31; N, 19.29%.

#### 3.1.6. Synthesis of Substituted-9*H*-pyrido[2,3-*b*]pyrrolizin-9-ylidenes (9c,f,g)

To a solution of 9*H*-pyrido[2,3-*b*]pyrrolizin-9-one **9a** (0.065 g, 0.38 mmol) in anhydrous ethanol (5 mL), the opportune heteroaryl carbohydrazide (0.38 mmol) was added. The resulting solution was heated to reflux for 24 h and then chilled overnight. The product was collected by filtration, washed with cold ethanol, and dried under vacuum to afford the desired compound **9f,g**, or purified by silica gel column chromatography using DCM:ethyl acetate, 75:25, as an eluent to give product **9c**.

*N′-[9H-Pyrido[2,3-b]pyrrolizin-9-ylidene]pyridine-3-carbohydrazide* (**9c**): Yield: 93%, yellow powder; m.p.: 182.5–182.7 °C; IR: 3385 (NH), 1684 (CO) cm^−1^; ^1^H-NMR (200 MHz, DMSO-*d*_6_) δ: 6.44 (1H, t, *J* = 3.1 Hz, H-7), 6.77 (1H, d, *J* = 3.1 Hz, H-8), 7.70–7.57 (3H, m, H-4′, H-5′ and H-6), 8.11 (1H, dd, *J* = 8.0, 1.2 Hz, H-3), 8.32 (1H, d, *J* = 8.0 Hz, H-4), 8.49 (1H, dd, *J* = 5.0, 1.2 Hz, H-2), 8.85 (1H, d, *J* = 4.1 Hz, H-6′), 9.13 (1H, s, H-2′), 13.92 (1H, bs, NH); ^13^C-NMR (50 MHz, DMSO-*d*_6_) δ: 108.2 (d), 115.3 (d), 115.5 (d), 117.9 (d), 118.0 (d), 119.2 (d), 123.9 (d), 125.9 (d), 128.5 (s), 129.6 (s), 135.8 (s), 144.0 (d), 145.5 (s), 146.4 (s), 148.8 (d), 161.0 (s). Anal. Calculated for C_16_H_11_N_5_O (MW: 289.29): C, 66.43; H, 3.83; N, 24.21%. Found: C, 66.28; H, 3.76; N, 24.11%.

*4-Amino-N′-[9H-pyrido[2,3-b]pyrrolizin-9-ylidene]benzohydrazide* (**9f**): Yield: 60%, yellow powder; m.p.: 276.1–277.0 °C; IR: 3447–3337 (NH_2_), 3227 (NH), 1662 (CO) cm^−1^; ^1^H-NMR (200 MHz, DMSO-*d*_6_) δ: 6.07 (2H, bs, NH_2_), 6.48 (1H, t, *J* = 3.4 Hz, H-7), 6.72 (3H, m, H-3′, H-5′ and H-3), 7.64 (2H, m, H-6 and H-8), 7.74 (2H, d, *J* = 8.6 Hz, H-2′ and H-6′), 8.15 (1H, dd, *J* = 8.1, 1.0 Hz, H-4), 8.56 (1H, dd, *J* = 5.0, 1.0 Hz, H-2), 13.73 (1H, bs, NH); ^13^C-NMR (50 MHz, DMSO-*d*_6_) δ: 106.7 (d), 113.1 (d × 2), 115.3 (d), 117.0 (d × 2), 118.5 (s), 118.9 (d), 125.3 (d), 129.1 (d), 130.1 (s), 135.4 (s), 136.5 (s), 143.9 (d), 146.2 (s), 152.9 (s), 162.3 (s). Anal. Calculated for C_17_H_13_N_5_O (MW: 303.32): C, 67.32; H, 4.32; N, 23.09%. Found: C, 67.45; H, 4.15; N, 23.31%.

*4-Hydroxy-N′-[9H-pyrido[2,3-b]pyrrolizin-9-ylidene]benzohydrazide* (**9g**): Yield: 90%, light yellow solid; m.p.: 294.5–295.3 °C; IR: 3415 (NH), 3201 (OH), 1675 (CO) cm^−1^; ^1^H-NMR (200 MHz, DMSO-*d*_6_) δ: 6.43 (1H, t, *J* = 3.7 Hz, H-7), 6.70 (1H, d, *J* = 3.7 Hz, H-8), 6.95 (2H, d, *J* = 8.8 Hz, H-3′ and H-5′), 7.60 (2H, m, H-4 and H-6), 7.85 (2H, d, *J* = 8.8 Hz, H-2′ and H-6′), 8.08 (1H, dd, *J* = 8.1, 1.3 Hz, H-3), 8.51 (1H, dd, *J* = 5.0, 1.3 Hz, H-2), 10.35 (1H, bs, OH),13.78 (1H, bs, NH); ^13^C-NMR (50 MHz, DMSO-*d*_6_) δ: 107.2 (d) 115.3 (d), 115.7 (d × 2), 117.3 (d), 118.9 (d), 123.0 (s), 125.5 (d × 2), 129.4 (d), 129.9 (s), 135.5 (s), 137.4 (s), 143.9 (d), 146.2 (s), 161.3 (s), 162.0 (s). Anal. Calculated for C_17_H_12_N_4_O_2_ (MW: 304.30): C, 67.10; H, 3.97; N, 18.41%. Found: C, 67.43; H, 3.85; N, 18.08%.

### 3.2. Biology

Tripentone derivatives, prepared as described above, were dissolved in dimethyl sulfoxide (DMSO) and then diluted in culture medium to obtain a DMSO concentration not exceeding 0.1%. HCT-116 (human colorectal carcinoma), MCF-7 (human breast cancer), and Caco-2 (human colorectal carcinoma) cell lines were purchased from American Type Culture Collection, Rockville, MD, USA and grown in Dulbecco’s Modified Eagle’s Medium(DMEM) supplemented with 10% fetal, 10% fetal bovine serum (FBS), penicillin (100 U/mL), streptomycin (100 μg/mL), and gentamicin (5 μg/mL). Cells were maintained in log phase by seeding twice a week at a density of 3 × 10^8^ cells/L in a humidified 5% CO_2_ atmosphere at 37 °C. In all experiments, HCT-116 and MCF-7 cells were left to incubate overnight to allow adhesion before treatment with the compounds or vehicle alone (control cells), while Caco-2 cells were treated 15 days after confluence, at which time the cells were differentiated in normal intestinal-like cells [38].

No differences were found between cells treated with DMSO 0.1% and untreated cells in terms of cell number and viability.

#### 3.2.1. Viability Assay In Vitro

Cytotoxic activity of the tripentone derivatives was determined by the colorimetric assay based on the reduction of 3-(4,5-dimethyl-2-thiazolyl)bromide-2,5-diphenyl-2*H*-tetrazolium (MTT) to purple formazan by mitochondrial dehydrogenases [39]. Briefly, HCT-116, MCF-7, and Caco-2 lines cells were seeded at 2 × 10^4^ cells/well in 96-well plates containing 200 μL DMEM. When appropriated, monolayer cultures were treated for 72 h with various concentrations (0.1–100 μM) of the tested compounds. Then cells were washed with fresh medium and 50 μL FBS-free medium containing 5 mg/mL MTT. Cells were incubated for 2 h at 37 °C, then the medium was discarded by centrifugation, formazan blue formed in the cells dissolved in DMSO, and absorbance was measured at 570 nm in a microplate reader (Bio-RAD, Hercules, CA, USA). Formazan of control cells was taken as 100% viability. The growth inhibition activity of compounds was defined as the GI_50_ value, which represents the log of the molar concentration of the compound that inhibits 50% cell growth. Each experiment was repeated three times in triplicate.

#### 3.2.2. Measurement of Phosphatidylserine (PS) Exposure

The apoptosis-induced PS externalization to the cell surface was measured using flow cytometry by double staining with Annexin V-Fluorescein isothiocyanate (Annexin V-FITC)/propidium iodide (PI). Annexin V binding to phosphatidylserine was used to identify the earliest stage of apoptosis. PI, which does not enter cells with intact membranes, was used to distinguish between early apoptotic cells (annexin V-FITC positive and PI negative), late apoptotic cells (annexin V-FITC/PI-double positive), and necrotic cells (annexin V-FITC negative and PI positive). After 24 h treatment, HCT-116 and MCF-7 cells were harvested by trypsinization and adjusted at 1.0 × 10^6^ cells/ mL with combining buffer according to the manufacturer’ instructions (eBioscience, San Diego, CA, USA). One hundred microliters of cell suspensions were added to a new tube, and incubated with Annexin V-FITC and PI solution at room temperature in the dark for 15 min. Then samples of at least 1.0 × 10^4^ cells were subjected to fluorescence-activated cell sorting (FACS) analysis by an Epics XL™ flow cytometer (Beckman Coulter, Fullerton, CA, USA) using Expo 32 ACD software (Beckman Coulter, Fullerton, CA, USA), using the appropriate bi-dimensional gating method.

#### 3.2.3. Cell Cycle Analysis

The cell cycle stage was analyzed by flow cytometry. HCT-116 and MCF-7 cells (5.0 × 10^4^ cells/cm^2^) were seeded in triplicate in 24-wells culture plates. After an overnight incubation, the cells were washed with fresh medium and incubated with compound **9h** in DMEM for 24 h. Then cells were harvested by trypsinization. Aliquots of 1.0 × 10^6^ cells were washed with PBS and incubated in the dark in a PBS solution containing 20 μg/mL PI and 200 μg/mL RNase, for 30 min at room temperature. Then samples of at least 1.0 × 10^4^ cells were subjected to FACS analysis.

## 4. Conclusions

In conclusion, eight new tripentones analogs were efficiently synthesized using a four-step sequence with fair overall yields (34–57%). All synthesized derivatives were tested for cytotoxicity against two human tumor cell lines, HCT-116 (human colorectal carcinoma) and MCF-7 (human breast cancer), by MTT assay. On the basis of the obtained results, the most active derivative was further investigated to study its mode of action. Flow cytometric analysis showed that it did not exert necrotic effects, but induced a clear shift of viable cells towards early apoptosis, causing the inhibition of the cell cycle progression in S and G2/M phases.

## Figures and Tables

**Table 1:** Tripentone derivatives **9a**–**h**.

Compound	R	Yield (%)
**9a**	-	70
**9b**	Pyridin-4-yl	69
**9c**	Pyridin-3-yl	93
**9d**	Furan-2-yl	72
**9e**	Thiophen-2-yl	70
**9f**	4-NH_2_-phenyl	60
**9g**	4-OH-phenyl	90
**9h**	Phenyl	76
